# Towards malaria elimination: a case–control study to assess associated factors to malaria relapses in the extra-Amazon Region of Brazil from 2008 to 2019

**DOI:** 10.1186/s12936-024-05133-4

**Published:** 2024-10-17

**Authors:** Klauss Kleydmann Sabino Garcia, Karina Medeiros de Deus Henriques, Antonio Alcirley da Silva Balieiro, Anielle de Pina-Costa, André M. Siqueira

**Affiliations:** 1 Faculty of Health Sciences, University of Brasilia, Federal District, Brasilia, Brazil; 2 Nucleus of Tropical Medicine, University of Brasilia, Federal District, Brasilia, Brazil; 3 Evandro Chagas National Institute of Infectious Diseases, Fundação Oswaldo Cruz (Fiocruz), Rio de Janeiro, Rio de Janeiro Brazil; 4 Instituto Leônidas E Maria Deane, Fundação Oswaldo Cruz (Fiocruz), Manaus, Brazil; 5 Fluminense Federal University, Niterói, Rio de Janeiro Brazil

**Keywords:** Malaria, Relapses, Epidemiological surveillance, Elimination, Case–control study

## Abstract

**Background:**

Malaria is an infectious disease caused by the *Plasmodium* species and is a global burden. When not treated correctly, it can reemerge as a relapse or recrudescence. Malaria relapse cases can contribute to maintaining active transmission chains and can influence the patient to develop severe malaria, potentially leading to hospitalization or death. The objective of this study is to estimate the number of malaria relapse cases in the extra-Amazon region of Brazil and to investigate the associated factors.

**Methods:**

This is a case–control study that analyses malaria infections caused by *Plasmodium vivax*, as reported in Notifiable Diseases Information System (Sinan) for the Brazilian extra-Amazon region (an area not endemic for the disease) from 2008 to 2019. For the identification of relapse cases, deduplication record linkage processes in R software were used. Malaria relapses were defined as the case group, and new malaria infections were defined as the control group. Logistic regression models were used to assess associated factors.

**Results:**

Of the 711 malaria relapses, 589 (82.8%) were first relapses. Most relapses (71.6%) occurred between 30 and 120 days after the previous infection. Malaria relapses are spread throughout the extra-Amazon region, with a higher concentration near big cities. Driver occupation was found to be a common risk factor compared to other occupations, along with asymptomatic individuals. Other associated factors were: being infected in the Brazilian Amazon region, having follow-ups for malaria relapses, and having parasite density of the previous infection higher than 10,000 parasites per mm^3^.

**Conclusions:**

This study provides evidence that allows malaria health surveillance services to direct their efforts to monitor cases of malaria in the highest risk segments identified in this study, particularly in the period between 30 and 120 days after being infected and treated. Relapses were associated to driver occupation, absence of symptoms, infection in endemic areas of Brazil, being detected through active surveillance or routine follow-up actions, and with parasitaemia greater than 10,000 parasites per mm^3^ in the previous infection. Improving cases follow-up is essential for preventing relapses.

**Supplementary Information:**

The online version contains supplementary material available at 10.1186/s12936-024-05133-4.

## Background

Malaria is an acute febrile infectious disease with potentially severe progression if left untreated or partially treated [1]. Globally, malaria is one of the most significant public health problems, and in Brazil, the Amazon region accounts for about 99% of the country’s cases, with the transmission of the disease being related to environmental and sociocultural conditions [2]. However, it is in the extra-Amazon region that malaria shows higher lethality due to diagnosis delay, either from inadequate clinical management of cases imported from endemic areas or even autochthonous cases in a few states [3].

The cases that occur in the extra-Amazon region are usually in the Atlantic Forest area [3]. The profile of cases imported means infection acquired from other countries or from states in the Brazilian Amazon region, with a high occurrence of cases caused by *Plasmodium falciparum* [4]—a species known to be associated with the development of severe forms of the disease [5, 6].

Because of faulty processes in the detection and treatment of malaria cases, new episodes (recurrences) of the disease may occur mainly due to a previous *vivax* infection [7]. Recurrences can account for up to 80% of the malaria burden in certain contexts. The factors that trigger the activation of *vivax* hypnozoites are not fully understood, and specific strain patterns, environmental factors, and host characteristics are potential contributors [8].

Recurrence after treatment leads to a new clinical episode with the risk of complications for the patient. Studies show that patients with recurrent *Plasmodium vivax* infections can become difficult to treat, and this condition can even be severe and fatal [9].

Thus, some terms are used to define repeated episodes of malaria in an individual. Malaria recurrences consist of recrudescence, relapse, or reinfection [10]. Reinfection is the occurrence of a new infection with parasites after a previous infection has been completely treated. It may be due to new mosquito bites and is often associated with living in endemic areas [11]. Recrudescence occurs with all *Plasmodium* species when blood-stage parasites are not completely eradicated and subsequently re-expand after the decline of drug concentrations in the blood [12]. Malaria relapse is defined as the recurrence of asexual parasitaemia following treatment of the disease, after its clearance has been confirmed, over varying periods of time [12]. Relapse occurs exclusively in *P. vivax* and *Plasmodium ovale*, usually when parasitaemia and clinical manifestations reappear due to the reactivation of dormant hypnozoites in the liver [12, 13].

Situations related to malaria relapses include therapeutic failures resulting from non or low adherence to treatment, parasite resistance to the drugs used, poor quality of the treatment provided, use of subtherapeutic doses of the drugs, reactivation of hypnozoites [13]. In Brazil, malaria treatment is available free of charge through the public health system and is provided immediately after diagnosis [14].

Thus, considering the full availability of treatment, it is necessary to understand which aspects are related to malaria relapses in this region of Brazil. These knowledge gaps hinder both the understanding of the epidemiological scenario of these relapses and their contribution to disease transmission, as well as complicate the design of public policies to prevent relapses.

Therefore, given that Brazil aims to eliminate malaria-related deaths by 2030 and eradicate autochthonous cases of the disease in its territory by 2035 [15], the objective of this study is to describe the epidemiological profile of malaria relapses in the extra-Amazon region of Brazil and analyse the factors associated with this event.

## Methods

### Study design, period and data sources

This is a case–control study that analyses malaria relapses caused by *P. vivax* infections. Mixed infections of *P. vivax* and other species were also included (*P. vivax* and *P. falciparum* or *P. ovale* or *Plasmodium malariae*). Malaria relapse cases were identified through analyses of malaria case databases from the Notifiable Diseases Information System (Sinan) from 2008 to 2019, provided by the Ministry of Health of Brazil in March 2021. Sinan receives notifications made in the extra-Amazon region, while the Epidemiological Surveillance Information System (Sivep-Malária) receives information from cases detected in the Amazon region [14].

### Study population, site and data sources

The study included data from malaria cases reported in Sinan in the extra-Amazon region of Brazil during the proposed period with a positive malaria test result, characterized by the presence of the parasite or any of its components in the blood.

The extra-Amazon region of Brazil comprises 4762 municipalities, with approximately 45,289,116 inhabitants, according to the Brazilian Institute of Geography and Statistics (IBGE) census in 2019. It consists of 17 states plus Federal District: Piauí, Ceará, Rio Grande do Norte, Paraíba, Pernambuco, Alagoas, Sergipe, Bahia, Mato Grosso do Sul, Goiás, Minas Gerais, Espírito Santo, Rio de Janeiro, São Paulo, Paraná, Santa Catarina, and Rio Grande do Sul [16].

### Record linkage process

Since every suspected case of malaria must be reported, and positive cases must undergo follow-up testing, the same individual may appear in the database more than once. It is also possible for the same individual to be reported more than once at different healthcare units, either due to the patient seeking care spontaneously or in cases where one healthcare unit reports a suspected case, and another healthcare unit reports the case when confirmed. Thus, the same individual may appear multiple times in the database due to duplicate reporting, referral, follow-up of malaria treatment (where relapses can be identified), or new infections.

To analyse the database, an identification system was prepared to recognize records belonging to the same individual. For this identification, the database was first pre-processed to prepare, organize, and structure it. Then, variables of interest were selected to identify paired records (matches), using commands in the R environment to verify which paired records referred to the same individual (deduplicated links) and post-processing with regrouping of records belonging to the same individual [17].

Initially, discrepancies and typographical errors in the variables “patient’s name,” “mother’s name,” and “date of birth” were identified and corrected, as they could compromise subsequent analyses [18]. The RLBigDataDedup function—of the RecordLinkage package—, which uses a probabilistic analysis approach, was applied to identify the same individual in the database [19]. The variables “patient’s name,” “mother’s name,” “date of birth,” and “sex” were used as matching variables [18]. The blocking technique was employed to reduce the number of comparisons between records by separating them into smaller blocks. Deduplication within a block involves running an algorithm that compares each record with others in the same block and determines whether they are duplicate pairs [20].

This processing in R Studio removes accents, excess whitespace, and prepositions that join first and last names, such as “DE,” “DAS,” “DA,” and “DO,” in the variables “patient’s name” and “mother’s name” [18, 21, 22]. A combination of three components represents the patient by first name, last name, and phonetic name. The soundexBR function returns the phonetic name as an alphanumeric code (soundex code) [23].

A selection of notification pairs identified as likely belonging to the same individuals was obtained through automatic verification, applying a probability threshold (probability > 0.6). The deduplication process R script is available in Additional file II. After this stage, a manual review was conducted to exclude incorrect pairs [24].

The use of deduplicate record linkage techniques, where individuals reported more than once were identified as malaria relapses, made the differentiation of malaria cases notifications between “new infections” and probable “relapses” possible.

In this study, the term “relapse” was used to define malaria infections that have re-emerged, either due to recrudescence or relapse [25]. For the comparison between groups, the deduplicated notifications were classified as “malaria relapses” (cases), and all other notifications were classified as new malaria infections (controls). Only relapse reports for episodes that occurred between 8 and 352 days after the first infection were considered [26]. Records found within 8 days after the previous infection were classified as duplicate reports—and, therefore, not included in the analysis—and records over 352 days after the previous infection were also not included due to difficulty in establishing whether these records were relapses or new infections.

### Variables of interest

The variables used for the study were: Notification date, Local of notification (states and municipalities), Date of first symptoms, presence of symptoms, Age, Sex, Pregnancy status, colour of skin, Education level, Residential zone, Type of detection, Probable place of infection, Occupation (main activity in the last 15 days), Parasite species, parasites density (mm^3^). Previous infections information was generated through the deduplication process.

### Statistical analyses

Descriptive analyses were conducted to characterize the relapse cases, with the presentation of the accumulated relapse rate calculated by dividing the total number of relapses during the period by the total number of new infections identified during the period, multiplied by 1000 patients.

After analysing the distribution of the variables, the chi-square test was used to identify which variables had a *p* value of less than 0.20 for subsequent inclusion in the univariate and multivariate logistic regression models. For the construction of the final model, the backwards method was used [27], retaining only the model with statistically significant variables and the best model adjustment parameters. Variables with a *p* value of less than 0.05 were considered statistically significant. To verify the final model fit, the Nagelkerke R square, Akaike information criterion (AIC), and Bayesian information criterion (BIC) were analysed.

For the statistical analysis processes, the following software was used: Microsoft Excel, R v. 4.3.3 [28], Jamovi v 2.3.13 [29], QGIS v. 3.22 [30].

### Ethics considerations

The data was requested from the Brazilian Ministry of Health through protocol 25209.000574/2020-42. The study was submitted to the Research Ethics Committee of the Evandro Chagas National Institute of Infectiology, and approved under Opinion: 4.264.823, CAAE: 35638420.3.0000.5262.

## Results

### Main findings

**Fig. 1:**
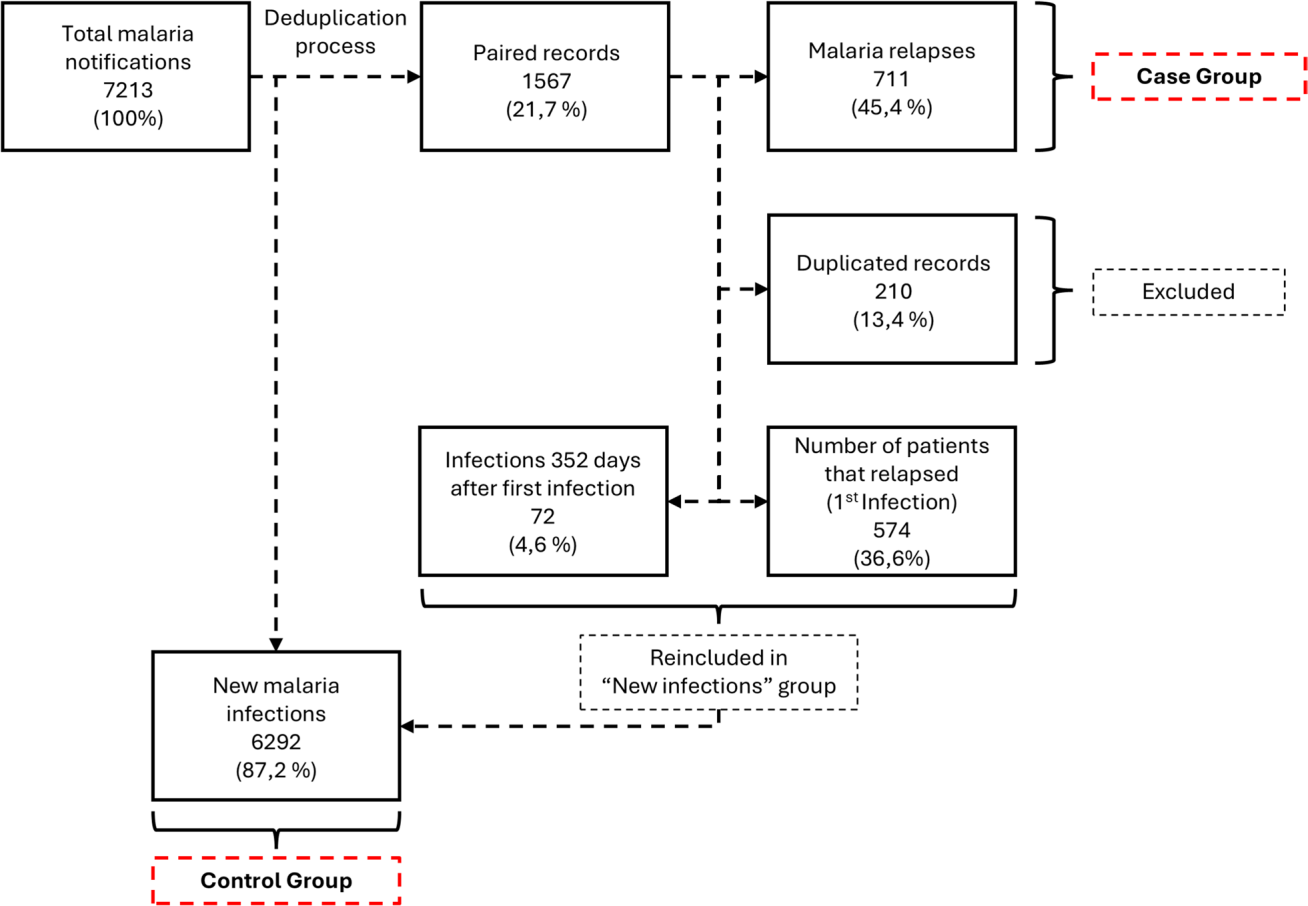
Flowchart of the malaria notification database from Sinan in the extra-Amazon region, from 2008 to 2019

Between 2008 and 2019, 7213 cases of malaria were reported in the extra-Amazon region. After the record linkage deduplication process, there were a total of 1567 (21.7%) paired records, of which 574 (36.6%) were patients being notified for their first malaria infection. These 574 patients generated 711 (45.4%) relapse episodes. Additionally, among the 1567 paired records, 210 (13.4%) were duplicated records—being excluded from the database. There were 72 (4.6%) relapses that occurred later than 352 days after the first infection, these records were reclassified as new infections. By the end there a total of 6292 new infections (control group) and 711 malaria relapses (case group) (Fig. 1).

**Fig. 2:**
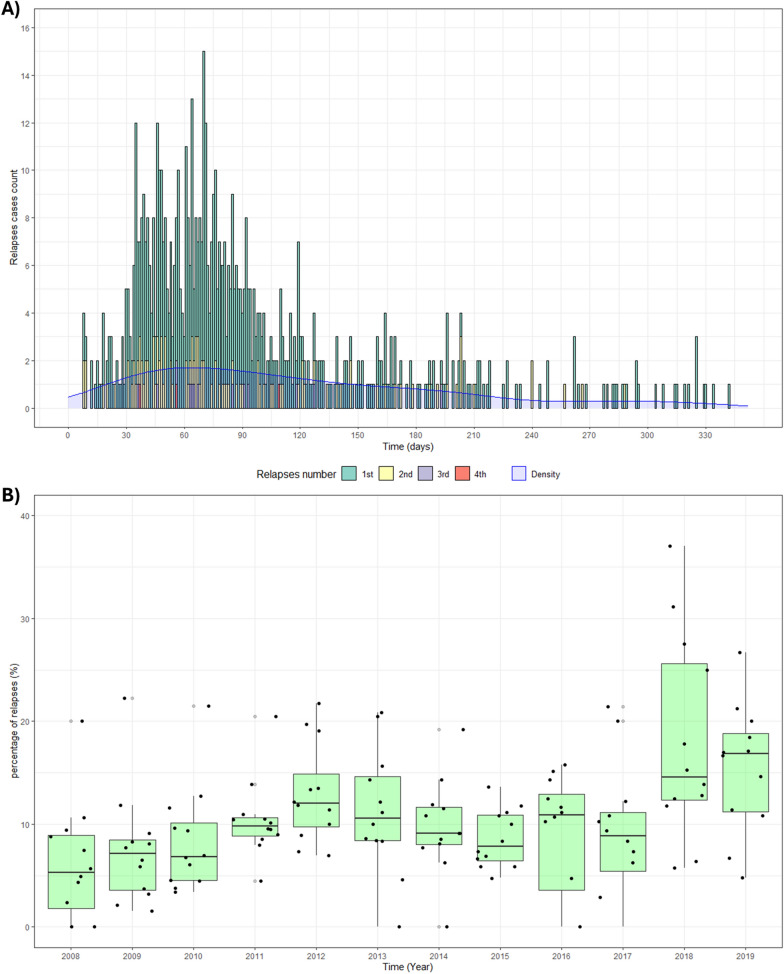
Distribution of malaria relapses cases over time. **A** Histogram of malaria relapse episodes; **B** distribution of malaria relapse cases proportion per month. *Source*: Sinan—Brazilian Ministry of Health

Of the 711 malaria relapses, 589 (82.8%) were a first relapse, 106 (14.9%) a second relapse of the same patient, 13 (1.8%) a third relapse, and 3 (0.4%) a fourth relapse. Most relapses in the time series were concentrated between 30 and 120 days (71.6%) after the previous infection, the mean was 94.4 days (standard deviation: 65.3) and then median was 75 days, (Q1 at 50 days; Q3 at 115 days) (Fig. 2A). The average annual proportion of relapses went from 5.2% in 2008 to 15.2% in 2019. The annual mean of occurrence was 10.1% with an accumulated relapse rate was 109.4 relapses per 1000 patients (Fig. 2B).

São Paulo is the state in the extra-Amazon region with the most notifications of malaria cases (new infections) accounting 1166 (18.5%) cases reported from 2008 to 2019, followed by Paraná (*N* = 653, 10.4%), Minas Gerais (*N* = 627, 10.0%), and Espírito Santo (*N* = 621, 9.9%).

**Fig. 3:**
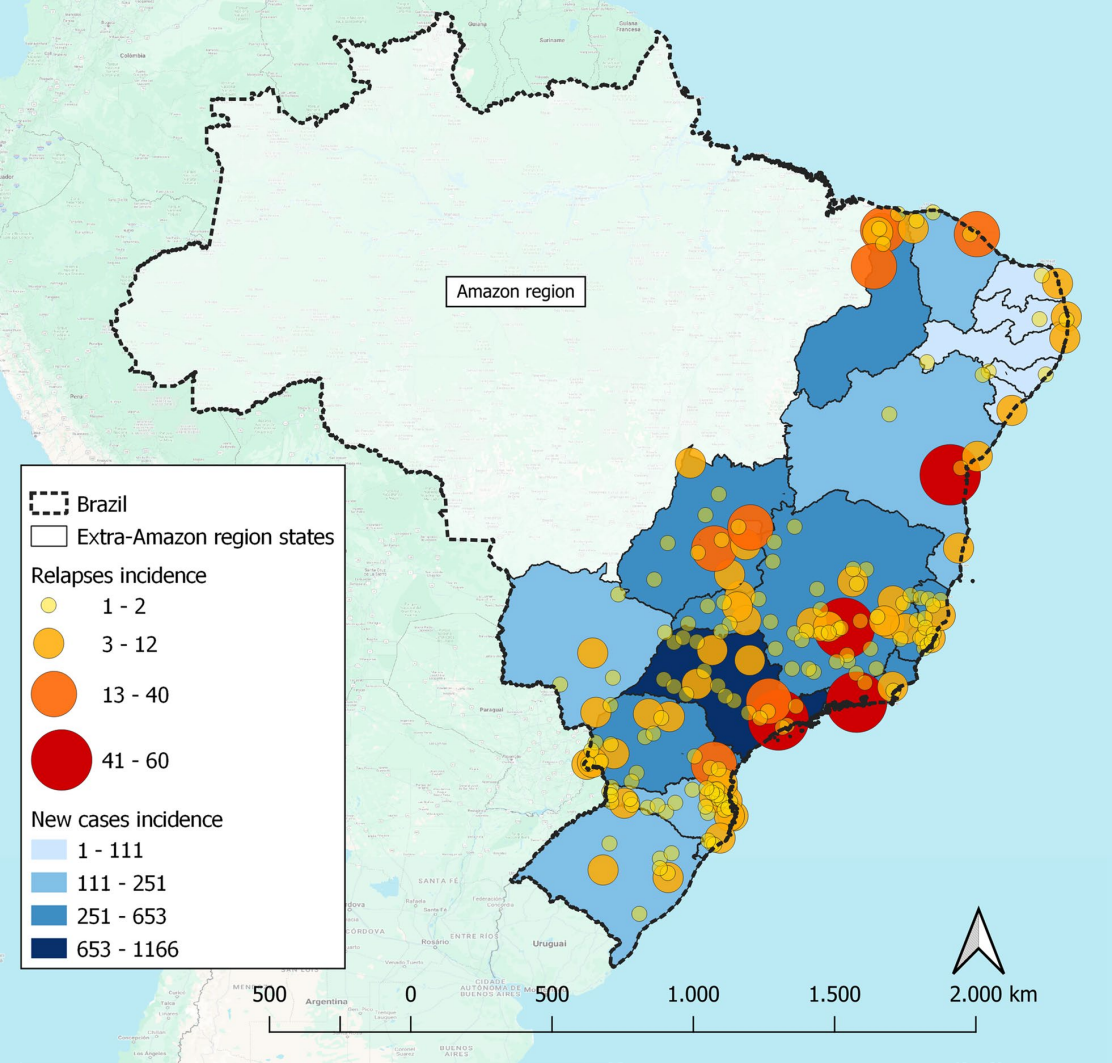
Distribution of accumulated new infections and relapses in the Brazilian extra-Amazonian region, 2008–2019. *Source*: Sinan—Brazilian Ministry of Health

Most occurrences of malaria relapses were in the state of Minas Gerais (*N* = 139; 19.5%) followed by São Paulo (*N* = 121; 17.0%). Top three cities with most relapses detection were all capitals: São Paulo/SP (*N* = 60, 8.4%), Belo Horizonte/MG (*N* = 49, 6.9%), and Rio de Janeiro/RJ (*N* = 48, 6.8%), followed by a spread across every extra-Amazon state, particularly in regions around the capitals (Fig. 3).

The epidemiological profile highlights malaria relapses among individuals of: male sex (83.0%), age between 20 and 39 (47.0%), white (48.1%) or black (41.3%) skin colour, education up to 11 years of education (53.9%), urban areas residency (79.6%), and travel related (17.4%), agricultural (14.8%) or undefined activities (34.2%). Most relapse cases presented symptoms (94.5%) and originated from infections that occurred in the Brazilian Amazonian region (68.5%). Only 5.5% of the relapses involved a mixed infection, only 18.0% of the cases were detected through active surveillance actions, and only 16.6% of the relapses had a previous infection with parasite density under 10,000 mm^3^ (Table 1).

**Table 1:** Epidemiological profile of new malaria infections and relapses reported in the Brazilian extra-Amazonian region, 2008–2019

Characteristics	Relapses (case group)	%	New infections (control group)	%	Total	%	*p* value (*X*^2^)
	711	10.2	6292	89.8	7003	100.0	
*Sex*							<0.001
Male	590	83.0	4910	78.0	5500	78.5	
Female	121	17.0	1382	22.0	1503	21.5	
*Age range*							0.272
00–04	15	2.1	132	2.1	147	2.1	
05–09	4	0.6	89	1.4	93	1.3	
10–19	38	5.3	409	6.5	447	6.4	
20–39	334	47.0	2969	47.2	3303	47.2	
40–59	248	34.9	2144	34.1	2392	34.2	
Over 60	72	10.1	549	8.7	621	8.9	
*Pregnancy*							0.072
Yes	3	0.4	43	0.7	46	0.7	
No	88	12.4	958	15.2	1046	14.9	
Not applicable	609	85.7	5140	81.7	5749	82.1	
Ignored/blank	11	1.5	151	2.4	162	2.3	
*Colour of skin*							0.216
White	342	48.1	2900	46.1	3242	46.3	
Black	308	43.3	2745	43.6	3053	43.6	
Yellow	2	0.3	55	0.9	57	0.8	
Indigenous	10	1.4	126	2.0	136	1.9	
Ignored/blank	49	6.9	466	7.4	515	7.4	
*Education*							0.439
Illiterate	4	0.6	86	1.4	90	1.3	
01–04 years of education	96	13.5	868	13.8	964	13.8	
05–08 years of education	133	18.7	1242	19.7	1375	19.6	
09–11 years of education	154	21.7	1311	20.8	1465	20.9	
Over 12 years of education	93	13.1	810	12.9	903	12.9	
Ignored/blank	231	32.5	1975	31.4	2206	31.5	
*Area of residency*							0.484
Urban	569	80	4933	78.4	5502	78.6	
Rural	115	16.2	1075	17.1	1190	17.0	
Ignored/blank	27	3.8	284	4.5	311	4.4	
*Occupation*							<0.001
Rural activities	105	14.8	1000	15.9	1105	15.8	
Construction of roads and dams	12	1.7	237	3.8	249	3.6	
Mining/gold mining	76	10.7	621	9.9	697	10.0	
Driver	81	11.4	433	6.9	514	7.3	
Tourism or travel	124	17.4	1570	25.0	1694	24.2	
Others	243	34.2	1897	30.1	2140	30.6	
Ignored/blank	70	9.8	534	8.5	604	8.6	
*Symptoms*							<0.001
Yes	672	94.5	6152	97.8	6824	97.4	
Assymptomatic	39	5.5	140	2.2	179	2.6	
*Local of infection*							0.006
AMZ autochthonous case	487	68.5	3954	62.8	4441	63.4	
Extra-AMZ autochthonous case	97	13.6	1133	18.0	1230	17.6	
Imported from other countries	85	12.0	784	12.5	869	12.4	
Ignored/blank	42	5.9	421	6.7	463	6.6	
*Type of detection*							<0.001
Active detection	128	18.0	1527	24.3	1655	23.6	
Passive detection	295	41.5	4331	68.8	4626	66.1	
LVC (follow-up for malaria relapse)	288	40.5	434	6.9	722	10.3	
*Species*							0.363
Mixed	39	5.5	400	6.4	439	6.3	
*P. vivax*	672	94.5	5892	93.6	6564	93.7	
*Parasite density from the previous infection*							0.005
Less than 10,000 mm^3^	593	83.4	5486	87.2	6079	86.8	
Over 10,000 mm^3^	118	16.6	806	12.8	924	13.2	

*Source*: Sinan—Brazilian Ministry of Health

The univariate analysis revealed statistically significant (*p* value <0.05) higher odds for malaria relapses among male individuals (COR: 1.37, 95% CI: 1.12–1.68), symptomless individuals (COR: 2.55, 95% CI: 1.77–3.67), individuals infected in the Amazonian region (COR: 1.44, 95% CI: 1.15–1.81), individuals examined for LVC (follow-up for malaria relapse) (COR: 9.74, 95% CI: 8.06–11.78), and individuals with previous infections with parasite density higher than 10,000 mm^3^ (COR: 1.35, 95% CI: 1.10–1.67). Occupations other than “Driver” were identified as protective factors against malaria relapses (Table 2).

**Table 2:** Logistic regression for predicting malaria relapses

	Univariate analysis model				Full model				Final adjusted model			
	COR	CI 95%		*p* value	AOR	CI 95%		*p* value	AOR	CI 95%		*p* value
*Sex (ref: female)*												
Male	1.37	1.12	1.68	0.002	1.26	0.74	2.16	0.397				
*Pregnancy (ref: not pregnant)*												
Yes	0.76	0.23	2.50	0.651	0.90	0.25	3.25	0.876				
Not applicable	1.29	1.02	1.63	0.033	1.01	0.56	1.81	0.972				
*Occupation (ref: driver)*												
Rural activities	0.56	0.41	0.77	<0.001	0.59	0.41	0.83	0.003	0.57	0.40	0.81	0.002
Mining	0.65	0.47	0.92	0.013	0.58	0.39	0.86	0.007	0.57	0.39	0.85	0.005
Tourism or travel	0.42	0.31	0.57	<0.001	0.46	0.33	0.64	<0.001	0.43	0.31	0.60	<0.001
Construction of roads and dams	0.27	0.15	0.51	<0.001	0.26	0.13	0.52	<0.001	0.26	0.13	0.52	<0.001
Others	0.69	0.52	0.90	0.006	0.75	0.55	1.02	0.065	0.70	0.51	0.95	0.021
*Symptoms (ref: with symptoms)*												
Assymptomatic	2.55	1.77	3.67	<0.001	2.01	1.28	3.17	0.003	1.99	1.26	3.13	0.003
*Local of infection (ref: extra-AMZ autochthonous case)*												
Imported from another country	1.27	0.93	1.72	0.129	1.21	0.83	1.76	0.314	1.21	0.83	1.74	0.321
AMZ autochthonous case	1.44	1.15	1.81	0.002	1.55	1.18	2.04	0.002	1.53	1.17	2.00	0.002
*Detection type (ref: passive detection)*												
Active detection	1.23	0.99	1.53	0.059	1.27	1.00	1.63	0.050	1.28	1.01	1.63	0.042
LVC (follow-up for malaria relapse)	9.74	8.06	11.78	<0.001	10.06	8.14	12.42	<0.001	10.30	8.35	12.70	<0.001
*Parasite density from the previous infection (ref: less than 10,000 mm*^*3*^*)*												
Over 10,000 mm^3^	1.35	1.10	1.67	0.005	1.37	1.06	1.75	0.015	1.35	1.06	1.74	0.017

Final model adjustment measures: AIC—3405; BIC—3486; *R*^2^—18.2%

*COR* crude odds-ratio, *AOR* adjusted odds-ratio, *ref* reference level

In the adjusted model, all occupations different from “Driver” continued to be identified as protective factors (*p* value <0.05). The variables that remained as risk factors (*p* value <0.05) included symptomless individuals (AOR: 1.99, 95% CI: 1.26–3.13), individuals infected in the Brazilian Amazon region (AOR: 1.53, 95% CI: 1.17–2.00), individuals tested for LVC (follow-up for malaria relapse) (AOR: 10.30, 95% CI: 8.35–12.7), and individuals with previous infections with parasite density higher than 10,000 mm^3^ (AOR: 1.35, 95% CI: 1.06–1.74). Additionally, active detected cases became statistically significant as a risk factor (AOR: 1.28, 95% CI: 1.01–1.63) (Table 2).

## Discussion

A total of 711 malaria relapses were identified, representing 9.9% of all malaria notifications in the period of study, with an average occurrence of 10.1% annually. Malaria relapses identification is concentrated in capitals cities or metropolises, probably due to access to bigger availability of health assistance services [31]. The relapses cases epidemiological profile is similar to the general malaria cases profile previously reported by Garcia et al. [4].

It is important to remember that 99% of malaria cases in Brazil are concentrated in the Amazon region [2], and that cases in the extra-Amazon region are usually from individuals infected either in the Brazilian Amazon or in other countries [4]. The occurrence of autochthonous cases in the extra-Amazon region is rare and generally associated with outbreaks in areas where the Atlantic Forest is present, along with the vector *Anopheles (Kerteszia) cruzii*, or with the presence of *Anopheles (Nyssorhynchus) darlingi*, the most prevalent vector in the Amazon. However, *An. darlingi* can occasionally be found in extra-Amazon regions, especially near forested environments and water bodies [3].

It has also been previously reported that malaria infection in the extra-Amazon region is linked to labor activities, such as those of drivers who travel between the extra-Amazon and Amazon regions [4], as well as miners working in the northern region of the country, who are later detected in the extra-Amazon region. This movement can affect the time it takes for patients to seek healthcare services after experiencing symptoms [32].

Thus, it is understood that in Brazil, the occurrence of malaria relapses is closely related to healthcare and health surveillance factors, such as proper case follow-up [3]. The treatment of *P. vivax* malaria in Brazil adheres to World Health Organization (WHO) recommendations, with chloroquine and primaquine being the mainstay of therapy [33]. Brazilian guidelines for uncomplicated *P. vivax* malaria treatment involves a combination of blood-stage and liver-stage therapies aimed at eliminating both active parasites and hypnozoites, including: chloroquine regimen for adults is administered at a dose of 25 mg/kg, spread over 3 days: day 1: 10 mg/kg, day 2: 10 mg/kg and day 3: 5 mg/kg, and primaquine, to prevent relapses by eliminating hypnozoites from the liver, prescribed alongside chloroquine. The standard primaquine dose is 0.5 mg/kg/day for 7 days. If the uncomplicated case relapses, the treatment is restarted with Primaquine combined with Artemether + Lumefantrine or Artesunate + Mefloquine [33].

Thus, it is understood that even with the free and immediate availability of treatment through the public health system, monitoring malaria cases can be challenging due to the patient’s occupational characteristics. The patient may travel, making follow-up difficult, and as a result, may not complete the treatment properly [3].

Most relapses happened between 30 and 120 days after the previous infection, evidencing that malaria relapses happen with higher frequency after 1 month of the initial infection—similar to what was reported in previous studies [25, 34].

Differently of what was reported in the study of Simões et al. [25], which was conducted in an endemic area. This study did not find sex, age and education as risk factor, although it did find relapses being more frequent among men, in people between 20 and 39 years old (in productive age) and with up to 11 years of study (elementary education).

Risk factors for malaria relapses such as high parasites density of first infection (higher than 10,000 mm^3^) was also reported by Simões et al. [25] in a study conducted in the endemic region of Brazil. Higher parasites density at the time of diagnosis of the first infection influencing on the occurrence of relapses could be related to lower levels of naturally acquired immunity [35], or to drug resistance or to higher concentration of dormant liver forms [13].

It was found that different occupations are protective factors for relapse when compared to the occupation of driver. Therefore, the occupation of driver is a common risk factor when compared to different occupations. In the Brazilian extra-Amazonian region, it is common to have men who travel with cargo transport (truck drivers) who transit between the Amazon region. This intense routine of travel and work can greatly influence the difficulty of keeping the treatment in progress and end up leading to its interruption and causing the relapse [36, 37]. Besides, the continuous flow of mobility may impact the search for medical care, and consequently the malaria diagnosis.

Also, as a risk factor for relapse, people were found who were previously infected in the Brazilian Amazon region, which is a vivax endemic region in Brazil. This factor may even be in line with the greater chance of relapse among drivers. Furthermore, previous studies have already reported traveling to endemic areas as a risk factor to malaria infection, therefore it can be well related to the relapse development [38].

Another risk factor for relapse was the absence of symptoms during relapse. Therefore, it is necessary to discuss the potential role of these people in maintaining transmission chains [39] or in the possibility of reintroducing malaria in receptive areas of the extra-Amazonian region [40, 41].

These relapses may not be detected because they do not present symptoms or maybe do not recognize the symptoms and consequently do not seek health services to restart treatment, thus, there is a greater chance of relapse among people who were tested precisely with the purpose of verifying whether there was a cure or not (LVC monitoring). It is understood here that if monitoring for LVC is carried out regularly and appropriately, the detection of relapses can be effective and contribute to the complete and adequate treatment of these cases [42]. If these driver workers are in permanent transit between regions of Brazil, in addition to the transit of asymptomatic people, these patients may also be influencing the maintenance of chains of infection in the Amazon region [43].

Although mixed infections have not been identified as a risk factor, it is important to highlight that when malaria is correctly diagnosed for mixed infections, it is possible to carry out appropriate treatment [44]. And the diagnosis of malaria only for the *P. vivax* species may not identify a mixed infection when carried out by microscopists with little experience—which may be a more common reality in the extra-Amazonian region considering that it is not an endemic region. Therefore, inadequate diagnosis can also influence the development of a relapse [3].

Due to the absence of a follow-up variable for cases in the malaria notification form in Sinan [22], the deduplicated record linkage strategy becomes one of the few plausible methods for identifying malaria recurrences for this purpose [17]. Considering that malaria is an acute disease [1] and that diagnosis and treatment in Brazil are free and provided by the National Health System [14], it is understandable to assume that identifying a case of malaria and providing treatment is sufficient for curing the patient. However, it is naïve not to pay attention to case follow-up, considering that incomplete treatment can lead to relapses and consequently severe cases of the disease [10].

Thus, it is suggested that a “progress” field be inserted into the epidemiological information systems for malaria, both for the extra-Amazon region of Brazil and the Amazon region. Despite being different systems (Sinan and Sivep-Malaria), they share extremely similar structures, and both present the same limitation. The insertion of this field will consequently strengthen the case follow-up process by epidemiological surveillance and provide a more qualified understanding of what happens to malaria cases after diagnosis and treatment are provided.

One of the challenges of this work, as with that of Simões et al. [25], is the inability to differentiate relapses from recrudescence. Therefore, it is suggested that other studies use molecular techniques to try to differentiate these types of relapses [45]. If effective, this strategy could be incorporated as a sentinel surveillance approach in areas with higher occurrences of relapses, such as the capitals of the extra-Amazon region.

Considering possible additional strategies that could be used to prevent malaria relapses, it is worth citing the use of tafenoquine for treating malaria cases in the extra-Amazon region [46, 47]. Tafenoquine is an anti-malarial medication used to treat and prevent malaria. It is particularly effective against *P. vivax*, a parasite responsible for recurring malaria infections. Tafenoquine works by targeting the dormant liver forms of the parasite, preventing malaria relapses, and is used for radical cure. Tafenoquine is also used for malaria prophylaxis in travelers and military personnel, protecting them from malaria infections, and it can be taken before, during, and after exposure to malaria-endemic areas [48].

Implementing tafenoquine in the extra-Amazon region of Brazil, where malaria is not endemic, could indeed be an interesting and potentially effective strategy, especially considering the severity and management challenges of malaria cases in such areas. It could potentially yield good results among people who frequently migrate between the endemic and non-endemic regions of Brazil [49, 50].

However, the need for Glucose-6-phosphate dehydrogenase (G6PD) testing is a challenge, which is mandatory due to the risk of hemolytic anemia when there is deficiency of this enzyme. Implementing widespread G6PD testing could be logistically challenging and costly to the health systems. Although, considering the low number of malaria cases in the extra-Amazon region—less than 1% of the total country cases—implementing the G6PD testing followed by tafenoquine treatment is feasible [51, 52].

### Study limitations

Despite the study not differentiating between relapse, reinfection, and recrudescence, it provides a scenario of relapse cases reported in the extra-Amazon region of Brazil. Additionally, there may be possible failures in the record linkage process and the number of matched records, especially when dealing with large databases [22]. However, the manual review of true pairs was a strategy to minimize the loss of true pairs [24].

As this is a retrospective study, individuals diagnosed with malaria before the study began may have experienced relapses during the analysed period and were considered new cases. It is also impossible to analyse a potential recurrence in individuals notified in endemic areas and reported to Sivep-malaria who may have been diagnosed with malaria in the Amazon region. To allow better identification of subjects in the database, a unique identifier would be ideal, differentiating various notifications of the same individual. The absence of this unique identifier in the Sinan complicates the recognition of relapses, potentially contributing to missed pairs [22]. These factors could have led to an underestimation of relapse cases in this study.

Recrudescence and relapses are difficult to differentiate clinically, particularly without molecular tests. Considering that this study used secondary data sources, it is not feasible to use such methods to differentiate types of recurrence.

Moreover, the quality of the data used could have influenced the results, even though the system from which the information was derived is robust and well-established for the country’s epidemiological surveillance. Also, underreporting of malaria cases and relapses in Sinan may have occurred due to limitations of the extra-amazon malaria epidemiological surveillance system. The lack of consistent case detection and follow-up could have impacted the identification of relapse cases.

## Conclusion

This study provides evidence that can help support malaria health surveillance services in the non-endemic regions of Brazil to improve their performance, particularly in the follow-up of malaria cases between 30 and 120 days after infection. Relapses were associated with occupations as drivers, absence of symptoms, infections acquired in endemic areas of Brazil, detection through active surveillance or routine follow-up actions, and parasitaemia greater than 10,000 parasites per mm^3^ in the previous infection.

Improving cases follow-up is an essential step for preventing relapses, interrupting transmission chains, and preventing severe forms of the disease, which can reduce hospitalizations and deaths. This process can contribute to achievement of Brazil’s elimination goals.

## Supplementary Information

**Additional file 1.** Simplified database repository.

**Additional file 2.** R script for deduplicate linkage.

## Data Availability

Sensitive information such as names and dates of birth are not available. Unidentified data is available in Additional file I.

## Abbreviations

AIC: Akaike information criterion
BIC: Bayesian information criterion
Sivep-Malária: Epidemiological Surveillance Information System
G6PD: Glucose-6-phosphate dehydrogenase deficiency
IBGE: Brazilian Institute of Geography and Statistics
LVC: Follow-up for malaria relapse
Sinan: Notifiable Diseases Information System
WHO: World Health Organization
