# Supplementary material for: Towards malaria elimination: a case–control study to assess associated factors to malaria relapses in the extra-Amazon Region of Brazil from 2008 to 2019
Source: Malar J. 2024 Oct 17;23:312. doi: 10.1186/s12936-024-05133-4 (PMC11488206; doi:10.1186/s12936-024-05133-4)
Supplement: Supplementary file 2 — Additional file 2. R script for deduplicate linkage. [file 12936_2024_5133_MOESM2_ESM.docx]

**Additional File II (R Script) - SCRIPT RECORD *LINKAGE***

## Packages required

library(tidyverse)

library(magrittr)

library(data.table)

library(RecordLinkage)

library(PPRL)

library(SoundexBR)

library(lubridate)

## importing df

BD <- fread("Sinan 2008-2019.csv",encoding = "Latin-1")

names(BD)

BD %>% select(NM_PACIENT) %>% view()

BD %>% select(NM_PACIENT) %>% view()

BD %>% group_by(RESULT) %>% summarise(n=n())

BD %>% group_by(NM_PACIENT) %>% summarise(n=n()) %>% view()

BD %>% filter(RESULT==4) %>% view()

# Data treatment

BD$NM_PACIENT1 <- BD$NM_PACIENT %>%str_squish() %>%

str_remove_all("[^[:alpha:][:space:]]") %>%

str_replace_all(" D..? ", " ") %>%

iconv(from = 'UTF-8', to = 'ASCII//TRANSLIT')

BD$NM_PACIENT1%>%

str_remove_all(":[punct]:")->BD$NM_PACIENT1

#BD %>% select(NM_PACIENT,NM_PACIENT1) %>% view()

BD$NM_PACIENT1[BD$NM_PACIENT1==""] <- NA

BD$NM_PACIENT1[BD$NM_PACIENT1=="SEM INFO"] <- NA

BD$NM_PACIENT1[BD$NM_PACIENT1=="SEMINFO"] <- NA

BD$NM_PACIENT1[BD$NM_PACIENT1=="NAO INFORMOU"] <- NA

BD$NM_PACIENT1[BD$NM_PACIENT1==""] <- NA

BD$NM_PACIENT1[BD$NM_PACIENT1=="NA"] <- NA

BD$DT_NASC[BD$DT_NASC==""] <- NA

BD$DT_NASC[BD$DT_NASC=="NA"] <- NA

str_squish(BD$NM_PACIENT1)->BD$NM_PACIENT1

gsub("\\b\\s?KG\\s?\\b","",BD$NM_PACIENT1)->BD$NM_PACIENT1

gsub("\\b\\s?TEL\\s?\\b","",BD$NM_PACIENT1)->BD$NM_PACIENT1

gsub("\\b\\s?FONE\\s?\\b","",BD$NM_PACIENT1)->BD$NM_PACIENT1

gsub("\\b\\s?JR\\s?\\b","",BD$NM_PACIENT1)->BD$NM_PACIENT1

gsub("\\b\\s?TL\\s?\\b","",BD$NM_PACIENT1)->BD$NM_PACIENT1

gsub("\\b\\s?TEM\\s?\\b","",BD$NM_PACIENT1)->BD$NM_PACIENT1

gsub("\\b\\s?NAO\\s?\\b","",BD$NM_PACIENT1)->BD$NM_PACIENT1

gsub("\\b\\s?TELEFONE\\s?\\b","",BD$NM_PACIENT1)->BD$NM_PACIENT1

gsub("\\b\\s?CELULAR\\s?\\b","",BD$NM_PACIENT1)->BD$NM_PACIENT1

gsub("\\b\\s?CELULA\\s?\\b","",BD$NM_PACIENT1)->BD$NM_PACIENT1

# Name, surname, last name

BD$PAC_1nome <- str_extract(BD$NM_PACIENT1, "[:alpha:]{1,}")

BD$PAC_2nome <- word(BD$NM_PACIENT1, 2)

BD$PAC_sobrenome <- sub(".* ", "",BD$NM_PACIENT1 )

BD$TamanhoDoNome<-sapply(strsplit(BD$NM_PACIENT1, " "), length)

# correcting repetition errors

ifelse(BD$TamanhoDoNome<="2",BD$PAC_2nome<-NA,

BD$PAC_2nome <- word(BD$NM_PACIENT1, 2))->BD$PAC_2nome

ifelse(BD$TamanhoDoNome<="1",BD$PAC_sobrenome<-NA,

BD$PAC_sobrenome <- sub(".* ", "",BD$NM_PACIENT1 ))->BD$PAC_sobrenome

BD %>% select(NM_PACIENT,NM_PACIENT1) %>% view()

BD %>% select(DT_NASC) %>% view()

# date of birth into day, month and year

BD$DIA <- BD$DT_NASC %>% str_sub(1,2)

BD$MES <- BD$DT_NASC %>% str_sub(4,5)

BD$ANO <- BD$DT_NASC %>% str_sub(7,10)

# treating mother’s name

BD$NM_MAE_PAC1 <- BD$NM_MAE_PAC %>% str_squish() %>%

str_remove_all("[^[:alpha:][:space:]]") %>% str_replace_all(" D..? ", " ") %>%

iconv(from = 'UTF-8', to = 'ASCII//TRANSLIT')

gsub("\\b\\s?-SIM-\\s?\\b","",BD$NM_MAE_PAC1)-> BD$NM_MAE_PAC1

BD$NM_MAE_PAC1 %>% str_remove_all("[special_string]")-> BD$NM_MAE_PAC1

BD$NM_MAE_PAC1[BD$NM_MAE_PAC1==""] <- NA

BD$NM_MAE_PAC1[BD$NM_MAE_PAC1==" "] <- NA

BD$NM_MAE_PAC1[BD$NM_MAE_PAC1=="SEM INFO"] <- NA

BD$NM_MAE_PAC1[BD$NM_MAE_PAC1=="SEMINFO"] <- NA

BD$NM_MAE_PAC1[BD$NM_MAE_PAC1=="SEM INFORMACAO"] <- NA

BD$NM_MAE_PAC1[BD$NM_MAE_PAC1=="SEM NOTIFICACAO"] <- NA

BD$NM_MAE_PAC1[BD$NM_MAE_PAC1=="SEM INFORMACAO"] <- NA

BD$NM_MAE_PAC1[BD$NM_MAE_PAC1=="SEM NFORMACAO"] <- NA

BD$NM_MAE_PAC1[BD$NM_MAE_PAC1=="SEM IFORMACAO"] <- NA

BD$NM_MAE_PAC1[BD$NM_MAE_PAC1=="SEM REGISTRO"] <- NA

BD$NM_MAE_PAC1[BD$NM_MAE_PAC1=="SEM REG"] <- NA

BD$NM_MAE_PAC1[BD$NM_MAE_PAC1=="SEMREGISTRO"] <- NA

BD$NM_MAE_PAC1[BD$NM_MAE_PAC1=="NAO INFORMOU"]<- NA

BD$NM_MAE_PAC1[BD$NM_MAE_PAC1=="NA"]<- NA

BD$NM_MAE_PAC1[BD$NM_MAE_PAC1=="NAO DECLARADA"]<- NA

BD$NM_MAE_PAC1[BD$NM_MAE_PAC1=="NAO COLOCARAM O NOME MAE"]<- NA

BD$NM_MAE_PAC1[BD$NM_MAE_PAC1=="NAO TEM"]<- NA

BD$NM_MAE_PAC1[BD$NM_MAE_PAC1=="NT"]<- NA

BD$NM_MAE_PAC1[BD$NM_MAE_PAC1=="NI"]<- NA

BD$NM_MAE_PAC1[BD$NM_MAE_PAC1=="NAO CLARADA"]<- NA

BD$NM_MAE_PAC1[BD$NM_MAE_PAC1=="NAO INFORMADO"]<- NA

BD$NM_MAE_PAC1[BD$NM_MAE_PAC1=="NAO COLOCARAM O NOME MAE"]<- NA

BD$NM_MAE_PAC1[BD$NM_MAE_PAC1=="OBS NAO TEM NOME MAE PACIENTE NA FICHA"]<- NA

BD$NM_MAE_PAC1[BD$NM_MAE_PAC1=="OBS NAO COLOCARAM NA FICHA"]<- NA

BD$NM_MAE_PAC1[BD$NM_MAE_PAC1=="OBS NAO CONSTA NA FICHA NOME MAE PACIENTE"]<- NA

BD$NM_MAE_PAC1[BD$NM_MAE_PAC1=="OBS NAO CONSTA O NOME MAE"]<- NA

BD$NM_MAE_PAC1[BD$NM_MAE_PAC1=="OBS NAO CONSTA O NOME MAE PACIENTE NA FICHA"]<- NA

BD$NM_MAE_PAC1[BD$NM_MAE_PAC1=="OBS NAO TEM NOME MAE NA FICHA"]<- NA

#############################################

BD$Mae_1nome <- str_extract(BD$NM_MAE_PAC1, "[:alpha:]{1,}")

BD$Mae_2nome <- word(BD$NM_MAE_PAC1, 2)

BD$Mae_sobrenome <- str_extract(BD$NM_MAE_PAC1, "[:alpha:]+$")

BD$TamanhoDoNmMae<-sapply(strsplit(BD$NM_MAE_PAC1, " "), length)

#####################################################

# correcting repetition errors

ifelse(BD$TamanhoDoNmMae<="2",BD$Mae_2nome<-NA,

BD$Mae_2nome <- word(BD$NM_MAE_PAC1, 2))->BD$Mae_2nome

ifelse(BD$TamanhoDoNmMae=="1",BD$Mae_sobrenome<-NA,

BD$Mae_sobrenome <- str_extract(BD$NM_MAE_PAC1, "[:alpha:]+$"))->BD$Mae_sobrenome

BD %>% select(NM_PACIENT,NM_PACIENT1,NM_MAE_PAC,NM_MAE_PAC1,DT_NASC,DT_NOTIFIC,ID_MUNICIP,NU_NOTIFIC) %>% view()

names(BD)

## Building subset of auxiliary variables

BDVX <- BD %>% filter(RESULT %in% c(4,5,6))

BDVX %>% select(NM_PACIENT,NM_PACIENT1,NM_MAE_PAC,NM_MAE_PAC1,RESULT,DT_NASC,DT_NOTIFIC,ID_MUNICIP,NU_NOTIFIC) %>% view()

BDVX %>% select(NU_IDADE_N) %>% view()

# comparing variables

matching_variables <- c("NM_PACIENT1", #1

"NM_MAE_PAC1", #2

"PAC_1nome", #3

"PAC_2nome", #4

"PAC_sobrenome", #5

"Mae_1nome", #6

"Mae_2nome", #7

"Mae_sobrenome", #8

"DIA","MES","ANO", #9,10,11

"CS_SEXO","NU_IDADE_N") #12,13

BDVX <- as.data.frame(BDVX)

df_names <- data.frame(BDVX[,matching_variables])

#df_names <- data.frame(BD_MAO %>% select(matching_variables))

threshold <- 0.5

matching_data <- df_names %>%

RLBigDataDedup(blockfld=list(1,c(3,4,5),2,c(09,10,11),12,13),exclude=c(6,7,8),phonetic = c(1,2,3,4,5)) %>%

epiWeights() %>% epiClassify(threshold) %>% getPairs(filter.link = "link", single.rows = TRUE)

matching_data %>% select(NM_PACIENT1.1,NM_MAE_PAC1.1,NM_PACIENT1.2,NM_MAE_PAC1.2,Class,Weight) %>% view()

write.csv(matching_data, 'matching_data.csv',row.names=FALSE)
